# Efficacy of meniscus suture absorbability on meniscus healing success rate via second-look arthroscopy after meniscal repair: a systematic review and meta-analysis

**DOI:** 10.1186/s12891-023-06602-8

**Published:** 2023-09-08

**Authors:** Wang Wei, Yi Zhang, Ruiying Li, Jianlong Ni, Dongjian Wang, Sanpeng Zhang, Zhibin Shi

**Affiliations:** 1 The First Department of Orthopaedics, The Second Affiliated Hospital of Xi’an Jiaotong University, Xi’an Jiaotong University, Xi’an, China; 2 Department of Orthopaedics, Tongji Hospital, School of Medicine, Tongji University, Shanghai, China; 3 Second Department of Orthopaedics, Shaanxi Sengong Hospital, Xi’an, China; 4 Department of Anesthesiology, The Second Affiliated Hospital of Xi’an Jiaotong University, Xi’an Jiaotong University, Xi’an, China

**Keywords:** Meniscus, Suture absorbability, Second-look arthroscopy, Meta-analysis

## Abstract

**Background:**

To preserve the meniscus’s function, repairing the torn meniscus has become a common understanding. After which, the search for the ideal suture material is continuous. However, it is still controversial about the efficacy of suture absorbability on meniscus healing.

**Methods:**

This review is designed according to the Preferred Reporting Items for Systematic Reviews and Meta-Analyses (PRISMA) guidelines. Inclusion criteria: (1) Studies on meniscus repair; (2) Second-look arthroscopy was performed; (3) The meniscus was repaired by absorbable and non-absorbable sutures; (4) The healing condition of repaired meniscus via second-look arthroscopy was described. Exclusion criteria: (1) Animal studies, cadaveric studies, or in vitro research; (2) Meniscus transplantation; (3) Open meniscus repair; (4) Reviews, meta-analysis, case reports, letters, and comments; (5) non-English studies. MEDLINE, Embase, and Cochrane Database were searched up to October 2022. Risk of bias and methodology quality of included literature were assessed according to ROBINS-I and the modified Coleman Methodological Scale (MCMS). Descriptive analysis was performed, and meta-analysis was completed by RevMan5.4.1.

**Results:**

Four studies were included in the systematic review. Among them, three studies were brought into the meta-analysis, including 1 cohort study and 2 case series studies about 130 patients with meniscal tears combined with anterior cruciate ligament injury. Forty-two cases were repaired by absorbable sutures, and 88 were repaired by non-absorbable sutures. Using the fixed effect model, there was a statistical difference in the healing success rate between the absorbable and the non-absorbable groups [RR1.20, 95%CI (1.03, 1.40)].

**Conclusion:**

In early and limited studies, insufficient evidence supports that non-absorbable sutures in meniscus repair surgery could improve meniscal healing success rate under second-look arthroscopy compared with absorbable sutures. In contrast, available data suggest that absorbable sutures have an advantage in meniscal healing.

**Trial registration:**

The review was registered in the PROSPERO System Review International Pre-Registration System (Registration number CRD42021283739).

## Background

The meniscus is an essential knee joint component for stability, load transmission, and articular cartilage protection. In order to preserve the function of the meniscus, repairing the torn meniscus has become a common understanding [17, 27]. Different repair methods developed according to different tear sites and types also significantly improved the clinical efficacy [4, 7, 10, 12, 14, 26]. However, the search for the ideal suture material is continuous, and numerous biomechanical studies have been performed on meniscal sutures of various materials. Whether the latest non-absorbable sutures has a higher meniscal healing success rate and whether it will cause more intraarticular damage than traditional absorbable sutures is the primary debate at present [1, 29]. Therefore, this study aimed to investigate the effect of meniscus suture absorbability on meniscus healing and hypothesized that non-absorbable sutures had a higher meniscal healing success rate than absorbable sutures. Second-look arthroscopy is an objective and reliable way to measure meniscus healing status. To accurately evaluate the difference in healing success rate for different suture materials, a systematic review and meta-analysis were conducted to evaluate the clinical efficacy of meniscus suture absorbability on meniscus healing success rate under second-look arthroscopy after meniscal repair.

## Materials and methods

This study was guided by the 2020 Preferred Reporting Items for Systematic Reviews and Meta-analyses (PRISMA) statement [16]. The review was registered in the PROSPERO System Review International Pre-Registration System (Registration number CRD42021283739).

### Research object

Inclusion criteria: (1) Studies on meniscus repair; (2) Second-look arthroscopy was performed; (3) The meniscus was repaired by absorbable and non-absorbable sutures; (4) The healing condition of repaired meniscus under second-look arthroscopy was described.

Exclusion criteria: (1) Animal studies, cadaveric studies, or in vitro research; (2) Meniscus transplantation; (3) Open meniscus repair; (4) Reviews, meta-analysis, case reports, letters, and comments; (5) non-English studies.

### Search strategy

With “meniscal” AND “second look”, “meniscal” AND “relook”, “meniscus” AND “second look”, “meniscus” AND “relook” as the search words, the databases MEDLINE, EMBASE, and Cochrane Database were searched by two independent authors (W.W.& Y.Z.) until October 2022.

### Data extraction and quality assessment

The article selection and quality assessment processes were independently performed by two authors with a pre-designed Excel form, and disagreement on study eligibility was solved by the senior author (Z.-B.S.). Extraction details contain demographic information, including patient cohort size and average age, time from injury to repair, and time from repair to second-look arthroscopy. Types and details of meniscal tears, methods of meniscal repair, suture materials, healing results via second-look arthroscopy, evaluation criteria for meniscus healing, cartilage condition, evaluation criteria for cartilage condition, level of evidence, complications and adverse events of suture materials were recorded if available. The quality of the included literature was assessed according to the modified Coleman Methodological Scale (MCMS), the categorical rating was considered to be excellent if the score was 85 to 100 points, good if it was 70 to 84 points, fair if it was 55 to 69 points, and poor if it was less 54 points [3]. And the evidence was graded according to the Levels of Evidence criteria published by the University of Oxford Centre for Evidence-Based Medicine [8, 31]. Each included study was independently scored by two authors (R.-Y.L. & J.-L.N.), and any discordant results were resolved by discussion until consensus was reached. Inter-rater reliability in assessment scoring was evaluated by calculating Fleiss κ values. The risk of bias was assessed by ROBINS-I [22].

### Study outcome and statistical analysis

The outcome of this study was the success rate of meniscus healing via second-look arthroscopy after the initial repair. Meta-analysis was performed using RevMan (Review Manager (RevMan) [Computer program]. Version 5.4.1, The Cochrane Collaboration, 2020.). Heterogeneity was tested using I^2^ metric with I^2^ > 25% as the cutoff for significant heterogeneity: a fixed-effect model was used when I^2^ < 25%; otherwise, a random-effect model was preferred. Relative risk (RR) was used as an efficacy analysis statistic, and each index was expressed with 95% Confidence Interval (CI). Subgroup analysis was performed based on suture materials. Sensitivity analysis was conducted.

## Results

### Overview of included studies

**Fig. 1:**
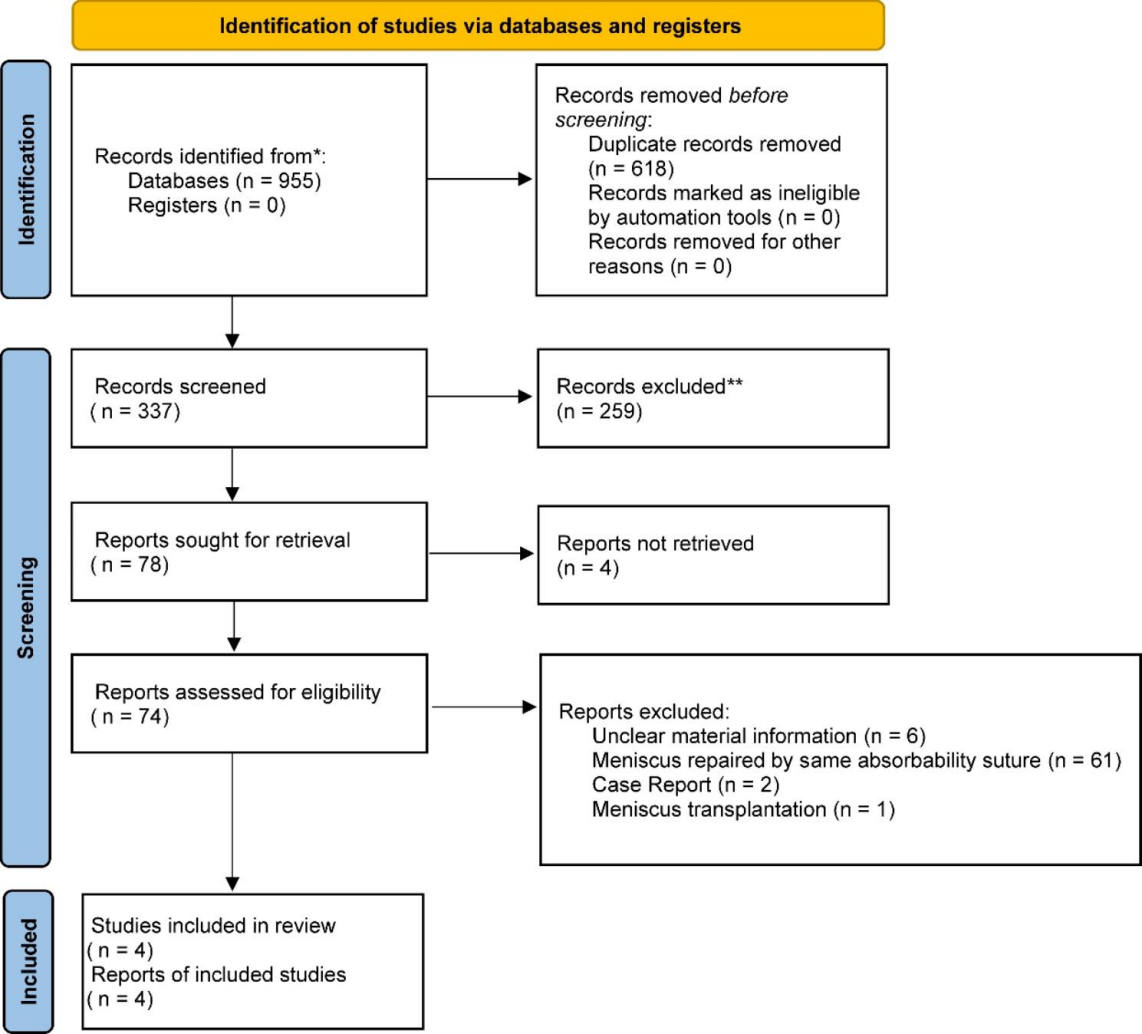
PRISMA flow diagram of systematic review

Nine hundred fifty-five related articles were detected. After the exclusion of duplicates, 337 articles remained. Two hundred fifty-nine studies were excluded by title and abstract screened, the remaining 78 papers were full-text assessed, and 74 papers that failed to meet the inclusion criteria were excluded. A total of four studies were included in the systematic review. Among them, one study was about meniscus tear combined with tibial plateau fracture [19], and three studies were about meniscus tear combined with ACL injury [5, 11, 21], including 1 cohort study and 2 case series studies, and were included in the meta-analysis (Fig. 1). The methodological quality of each study was assessed according to the modified Coleman Methodological Scale (MCMS) score [3]; two articles ranged between 55 and 69 [11, 21], and two articles were less than 54 [5, 19]. The risk of bias assessed by ROBINS-I showed a serious risk of bias. A total of 187 eligible patients who underwent second-look arthroscopy after initial meniscus repair were included in this study (Table 1).

**Table 1:** Information of Included Literature

Study		Seo et al. [21]	Miao et al. [11]	Feng et al. [5]	Ruiz-Ibán et al. [19]
Study Design		case series	cohort study	case series	case series
Evidence grade		IV	II	IV	IV
Second-look Cases		61	89	67	13
		PDS:28 ULTRABRAID:33	Arrow:43 ULTRABRAID:35 PDS:11	PDS:3 PDS + Ethibond:40 Ethibond:24	PDS:4 PDS + ULTRABRAID:2 ULTRABRAID:7
Mean age, y^a^		PDS:31.0 ± 10.4 Fast-Fix:29.4 ± 8.6	25.4 ± 7.7	25, 14–47	47.5 ± 13.7
Mean time^b^	Injury-surgery	PDS:32.3 ± 27.6 d Fast-Fix:26.6 ± 14.9 d	14.0 ± 30.0 m	26 m,7 d-19 y,7 m	-
	Surgery-Sec Look	PDS:16.0 m Fast-Fix:16.5 m	25.4 ± 6.0 m	25 m, 14-66 m	14.2 ± 10.1 m
Case information	Case type	Meniscus tear ACL injury	Meniscus tear ACL injury	Meniscus tear ACL injury	Meniscus tear tibial plateau fracture
	Medial/Lateral	39 Medial:22 Lateral	65 Medial:24 Lateral	60 Medial:7 Lateral	12 Lateral:1 Medial
	Site^c^	PH	PH M AH-PH M-PH	-	2 AH 1 M 3 AH-M 5 M-PH 2 AH-M-PH
	Type	Longitudinal	Longitudinal	Banket handle	12 Longitudinal 1 Radial
	Mean tear length, mm	PDS:14.2 ± 2.4 ULTRABRAID:14.0 ± 2.8	19.6 ± 7.6	-	-
Treatment	Repair method	all-inside hook all-inside FasT-Fix	outside-in all-inside FasT-Fix	all-inside hook inside-out	outside-in all-inside FasT-Fix
	Extra procedure^d^	ACL-R	ACL-R	ACL-R	Fracture reduction Internal fixation
Rehabilitation		0-3 W Partial weight-bearing Flexion 30° 3 W Flexion increased 6 W Full weight-bearing 10 M Sports activity	0-6 W Flexion 120° 6-7 W Flexion increased 6-7 W Full weight-bearing 6-12 M Sports activity	0-4 W Flexion 90° 4 W Partial weight-bearing 6 W Full weight-bearing 10 M Sports activity	0-3 W Flexion 90° 6 W Flexion 140° 0-8 W No weight-bearing 12 W Full weight-bearing

^a^Mean age y: year(s); ^b^Mean time m: month(s); d: day(s); ^c^The location AH: Anterior horn; PH: Posterior horn; M: Midbody; ^d^ACL-R: anterior cruciate ligament reconstruction

### Effect of suture absorbability on meniscus healing via second-look arthroscopy

#### Suture material

A total of four studies were included, each of which used absorbable and non-absorbable sutures to repair torn meniscus, respectively. Ruiz-iban et al. [19] and Miao et al. [11] use absorbable polydioxanone suture (PDS; Ethicon, Somerville, New Jersey) to repair the torn meniscus by outside-in method and all-inside Fast-Fix device (Fastfix; Smith & Nephew, Andover, Massachusetts) loaded non-absorbable ULTRABRAID ultra-high molecular weight polyethylene (UHMWPE) suture to repair meniscal tears. Feng et al. [5] repair the meniscus by inside-out method using non-absorbable Ethibond Suture (Ethicon, Somerville, NJ) and uses all-inside Suture Hook (Suture Hook CorkScrew; Linvatec, Largo, FL) loaded absorbable polydioxanone suture (PDS; Ethicon) to repair meniscal tears; Seo et al. [21] use absorbable polydioxanone sutures (PDS; Ethicon, Somerville, NJ) loaded by suture hook (Linvatec, Largo, FL) and Fast-Fix device loaded non-absorbable ULTRABRAID (Smith & Nephew Endoscopy, Andover, MA) to repair the torn meniscus.

### Meniscus healing status under second-look arthroscopy

Among the four studies included in this research, two studies utilized the previous evaluation criteria of meniscal healing under second-look arthroscopy, which were Morgan [13] criteria used by Feng et al. [5], Scott [20] criteria used by Seo et al. [21], and the other two studies [11, 19] used their criteria. The meniscus healing evaluation criteria used in the four studies were all based on the integrity evaluation of repaired meniscus. According to the stability of repaired meniscus, whether there was a residual tear, the proportion, thickness, width of the residual tear, and whether there was a re-tear, meniscus healing conditions can be divided into complete healing, incomplete healing, failure, or complete healing and non-healing. The success rate of meniscus healing via second-look arthroscopy refers to the proportion of complete and incomplete healing to total meniscus repair cases. The meniscus healing status of included studies was evaluated based on the integrity of the meniscus repaired site via second-look arthroscopy, so the success rate could be used to evaluate the meniscus healing status.

The subjects receiving absorbable polydioxanone suture repair in all studies were compared against control groups either receiving UHMWPE suture repair or polyester suture repair at the time of initial surgery (Table 2). In order to exclude the influence of combined repair with different suture materials, this study only analyzed the data observed via second-look arthroscopy after initial repair with single suture material in the included studies. Three studies evaluated the effect of different suture absorptivity on meniscal repair combined with anterior cruciate ligament reconstruction. Seo et al. [21] showed that there was no statistical significance in the healing of meniscus repaired by absorbable suture and non-absorbable suture in red-red and red-white zones, respectively (red-red zone, P = 0.692; red-white zone, P = 0.293), but the overall healing status showed that absorbable suture was significantly better than non-absorbable suture (P = 0.048). In the PDS suture group, there were 23 cases (82.1%) of complete healing, 4 cases (14.3%) of incomplete healing, and 1 case (3.6%) of failure. In the Fast-Fix suture group, there were 18 cases (54.5%) of complete healing, 8 cases (24.2%) of incomplete healing, and 7 cases (21.2%) of failure. The overall healing success rate was 96.4% and 78.8%, respectively. Miao et al. [11] show no statistical significance in the healing status between groups of different suture materials (P = 0.706). Feng et al. [5] compared the effects of polydioxanone and polyester sutures on bucket-handle tears combined with ACL injury. This study showed no statistically significant difference in the healing between the absorbable PDS suture and the non-absorbable Ethibond suture (P = 1.000). Ruiz-Iban et al. [19] researched meniscus tears with tibial plateau fractures. The study shows that 4 cases were healed by absorbable PDS sutures, 6 cases were healed, and 1 case was partially healed by non-absorbable UHMWPE suture.

**Table 2:** Success rate via Second-look arthroscopy

		Absorbable	Non-absorbable
Seo et al. [21]	Material	polydioxanone (PDS)	UHMWPE (ULTRABRAID)
	Method	All-inside (Hook)	All-inside (Fast-Fix)
	Healed (Total)	27(28)	26(33)
	Success rate	96.4%	78.8%
Miao et al. [11]	Material	polydioxanone (PDS)	UHMWPE (ULTRABRAID)
	Method	Outside-in	All-inside (Fast-Fix)
	Healed (Total)	10(11)	28(35)
	Success rate	90.9%	80.0%
Feng et al. [5]	Material	polydioxanone (PDS)	polyester (ETHIBOND)
	Method	All-inside (Hook)	Inside-out
	Healed (Total)	3(3)	20(24)
	Success rate	100%	83.3%
Ruiz-Ibán et al. [19]	Material	polydioxanone (PDS)	UHMWPE (ULTRABRAID)
	Method	Outside-in	All-inside (Fast-Fix)
	Healed (Total)	4(4)	7(7)
	Success rate	100%	100%

### Cartilage damage

Ruiz-iban et al. [19] evaluated the cartilage condition of patients who underwent initial meniscus repair combined with reduction and internal fixation of tibial plateau fracture via second-look arthroscopy; the results showed that the cartilage surface condition of all compartments unaffected by fracture did not change under second-look arthroscopy. Among the four patients with meniscus repaired by absorbable PDS suture, there was no change in cartilage condition, nine patients with meniscus repaired by Fast-Fix non-absorbable sutures, there were no changes in cartilage in 6 cases, two patients were lost to follow-up, and one patient with Schatzker type 1 combined with the banket-handle tear of the lateral meniscus was sutured four times by Fast-Fix. A 1cm^2^ ICRS II lesion was found at the lateral femoral condyle during second-look arthroscopy 26.9 months later.

### Meta-analysis

Three studies about meniscus tears combined with ACL injury [5, 11, 21] were included in the meta-analysis. There was no statistical heterogeneity among studies (P = 0.68, I^2^ = 0%); the heterogeneity between literature was mainly attributed to the types of meniscus injury and meniscus repair methods in each study. It is unsuitable for evaluating publication bias due to the limited included literature.

Two studies [11, 21] reported the meniscal healing rate of 87 patients with absorbable polydioxanone sutures and non-absorbable UHMWPE sutures via second-look arthroscopy. Among them, 39 cases were treated with absorbable PDS sutures, and 64 were treated with non-absorbable ULTRABRAID sutures. There was no statistical heterogeneity among the studies (P = 0.50, I^2^ = 0%). The meta-analysis results demonstrated that the meniscus healing rate of the group with absorbable polydioxanone suture significantly differed from those with non-absorbable UHMWPE suture under second-look arthroscopy [RR1.22, 95%CI (1.03, 1.44)]. The results showed that the meniscus healing success rate of the absorbable group was higher than the non-absorbable group.

Feng et al. [5] reported the meniscus healing rate of 23 patients with absorbable polydioxanone and non-absorbable polyester sutures under second-look arthroscopy. Among them, 3 cases were treated with absorbable PDS sutures, and 20 were treated with non-absorbable Ethicon sutures. The results found no significant difference in meniscal healing rate between different suture groups via second-look arthroscopy after initial meniscal repairing [RR1.07, 95%CI (0.71, 1.61)].

**Fig. 2:**
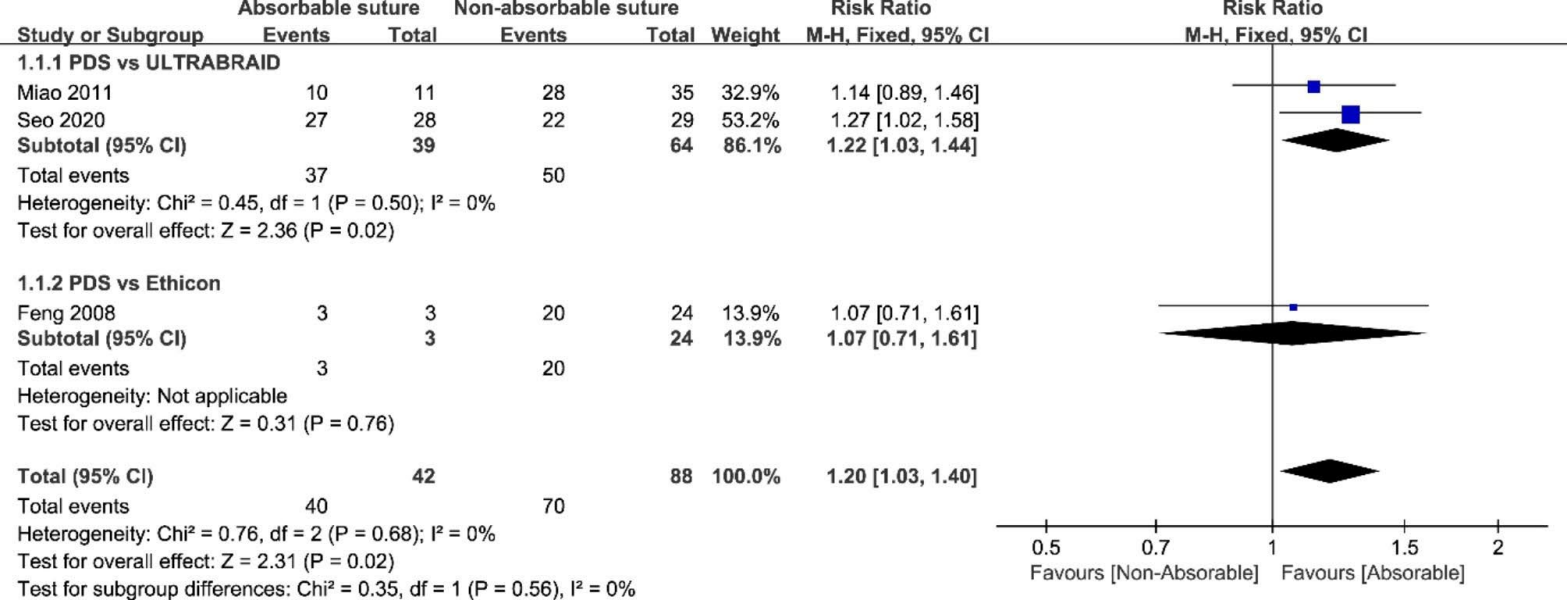
Results of the meta-analysis for the different suture groups

Combining all subgroups, the cases of meniscus healing rate under second-look arthroscopy after meniscus repair combined with ACL reconstruction was summed up to 130 patients. Among them, 42 cases were treated with absorbable sutures, and 88 were treated with non-absorbable sutures. There was no statistical heterogeneity among the subgroups (P = 0.68, I^2^ = 0%), and the differences in meniscus healing rate were statistically significant in the fixed-effect model [RR1.20, 95%CI (1.03, 1.40)]. The results showed that the success rate of meniscus healing under second-look arthroscopy was higher in the group with absorbable sutures than in the group with non-absorbable sutures (Fig. 2).

Sensitivity analysis was performed by changing the effect model and excluding individual references. The combined results were not statistically significant when data from Seo et al. [21] was excluded [RR1.12, 95%CI (0.90, 1.38)]. Changing the effect model or excluding the other two studies did not significantly affect the overall results.

## Discussion

Meniscus repair has undergone several technological updates, from the early open meniscal suturing to arthroscopic meniscal repair methods such as inside-out, outside-in, and all-inside. The all-inside repairs were also upgraded from the first-generation meniscus suture hooks [12], the second-generation T-FIX (Smith & Nephew, Andover, MA, USA) repair device loaded with non-absorbable polyester suture and polyethylene bar, and the third generation including meniscal arrows, darts, screws, and staples. Most are made of rigid poly-l-lactic-acid (PLLA) and variants of a faster resorbing copolymer, 80 L/20D, L PLA. PLLA can maintain the strength for 12 months and need over 2–3 years for complete reabsorption. The faster reabsorption copolymer (80 L/20D, L PLA) retains its strength for up to 24 weeks and then gradually reabsorption [30]. The fourth generation is flexible, suture-based meniscal repair devices that achieve variable compression and re-tensioning across the tear [24]. Currently, the most common repair methods for meniscus tears are the inside-out method by double-armed needles, the outside-in method by curved or straight needles, and the fourth-generation all-inside meniscal repair devices [10, 12, 14, 26], which all rely on sutures to provide tension on tears to promote meniscus healing. However, it is still controversial whether non-absorbable sutures have a higher meniscal healing success rate than absorbable sutures. Meniscus healing generally takes several months, and the absorbable meniscus sutures are mostly made of polydioxanone (PDS), polyglycolic acid (Dexon), and polyglactin-910 (Vicryl); degradation of the material will occur at 3-6 weeks postoperatively, resulting in a decrease in strength. It is believed that premature degradation will lead to loss of suture tension and decreased support for the torn area, thus affecting meniscus healing. Nowadays, non-absorbable sutures are mostly made of ultra-high molecular weight polyethylene (UHMWPE), which can maintain tension at the suture site for a long time and promote meniscus healing. However, the view was expressed that the non-absorbable knot left at the meniscus surface will damage articular cartilage and accelerate the progression of osteoarthritis.

At present, some novel meniscus suture products have been designed and come into the market. The mainstream is based on ultra-high molecular weight polyethylene (UHMWPE) suture material, such as FiberWire (Arthrex) and ULTRABRAID (Smith & Nephew Endoscopy). In addition, DePuy Mitek promotes the partially absorbable suture ORTHOCORD (DePuy Mitek), which is woven from 55% PDS & 45% high molecular weight polyethylene, can leave more minor suture knots after surgery, theoretically reducing the wear of suture knots to surrounding tissues. This partially absorbable suture allows more minor knots left in situ after surgery, reducing friction between knot and tissue. Besides, DYNACORD (DePuy Mitek) consists of a high-molecular weight polyethylene, polyester, and nylon braided sheath with a silicon and sodium chloride core that expands radially and shrinks axially in the liquid. This adaptive property keeps the repair structure stable, reduces suture relaxation, and prevents the formation of suture interspace. MaxBraid (Zimmer Biomet) is made of UHMWPE, which has no core design that allows the suture to be flat when tied, reducing the size without reducing the strength of the knots. Moreover, a range of flat sutures are currently being promoted, for example, ULTRATAPE (Smith & Nephew Endoscopy), PERMATAPE (DePuy Mitek), SutureTape (Arthrex), XBraid (Stryker), Hi-Fi Tape (Conmed), BROADBAND (Zimmer Biomet) and others. They are all woven from UHMWPE, and the wider contact area can reduce the distribution pressure at the repair site; a wider suture structure can reduce the possibility of tendon tissue being cut by suture and provide smaller knot volume when tying. These ultra-high molecular weight polyethylene meniscal sutures have higher breaking strength, better smooth properties, and better wear resistance than conventional polyester sutures. These new sutures are designed to improve healing rates after meniscus sutures and reduce suture side effects such as knot reaction, cartilage damage, and suture cutting. However, due to the lack of high-quality, long-term randomized controlled trials, it is still unsure whether there is a real improvement.

Based on this issue, we expected to use meta-analysis to find relatively objective evidence from the limited available literature to guide current clinical practice. Therefore, we hypothesized that non-absorbable sutures had a higher meniscal healing success rate than absorbable sutures. In our research, three studies [5, 11, 21] reported the meniscus healing success rate via second-look arthroscopy after meniscus repair with different absorbability sutures. Of a total of 130 patients, 42 cases were treated with absorbable sutures, and 88 cases were treated with non-absorbable sutures; the differences in combined meniscus healing rate were statistically significant [RR1.20, 95%CI (1.03, 1.40)], indicating that the success rate of meniscus healing under second-look arthroscopy in the absorbable suture group was better than that in the non-absorbable suture group.

Meanwhile, several studies have pointed out that the permanent knot of non-absorbable sutures may cause cartilage damage, synovium irritation, and meniscus cysts [9, 15, 23]. Yoo et al. [28] reported a case about all-inside meniscus repair with non-absorbable Ethibond suture combined ACL reconstruction. Cartilage damage was found on the patella and lateral femoral condyle surface during second-look arthroscopy. Gliatis et al. [6] found cartilage damage adjacent to the anchor implantation point after all-inside repair by RapidLoc (Mitek Surgical Products, Westwood, MA, USA) via second-look arthroscopy. However, no direct evidence of knot-caused articular cartilage damage was found in this study. In contrast, some studies [2, 18, 25] suggest that iatrogenic cartilage damage is common during arthroscopy. It means that the cartilage damage observed under second-look arthroscopy has multiple causes. It may be related to non-absorbable suture knot stimulation, anchor injury of the meniscus repair device, or iatrogenic injury during primary arthroscopy. To verify the relationship between non-absorbable suture knots and cartilage damage, it is necessary to design a study about the cartilage condition after non-absorbable sutures repair the isolated meniscus tear.

In this study, several major databases were searched to identify existing literature that used absorbable and non-absorbable sutures for meniscus repair in the same study and evaluated meniscus status by second-look arthroscopy. It was found that there are only a few studies that compare suture materials with different absorbability and use second-look arthroscopy to evaluate the healing effect at present. The robustness and representativeness of these study results may be affected by the lack of comparative studies on meniscus suture materials and the insufficient case data of second-look arthroscopy. However, the meniscus suture material is an issue that has attracted much attention. With the popularization of the meniscus repairing concept and the improvement of arthroscopy technology, this problem has become more prominent and urgent. With the help of meta-analysis, we can summarize the existing limited evidence and draw a relatively objective conclusion, which can be used as evidence to provide clinical suggestions with certain reference values for current meniscus repairing and provide direction for future suture material research. Further high-quality studies are needed to compare the healing effects of sutures with different absorbability on meniscus tears.

### Limitations

In general, the meniscus healing success rate may be related to the influence of device structure which loaded sutures, the suture material, and the persistent stress changes. Because second-look arthroscopy is a reliable but invasive method to evaluate the meniscus healing status objectively, there are few comparative studies on meniscus healing rate of different suture materials via second-look arthroscopy that can be retrieved in the database, and no high-quality randomized controlled trials have been found to address this issue. Therefore, this review is limited by the number of available studies. The quantity and quality of researches included in this study are mediocre; no in-depth analysis of demographic, repair methods, suture diameter, meniscus tear types, and other factors were conducted. In addition, there is a certain publication bias because the literature source is only in English. These factors may lead to bias in the results. Therefore, this evidence should be used cautiously.

## Conclusion

In early and limited studies, there is insufficient evidence to support that the usage of non-absorbable sutures during meniscal repair could improve meniscal healing success rate under second-look arthroscopy compared with the usage of absorbable sutures. On the contrary, according to the limited evidence, absorbable sutures seem to have the advantage in meniscal healing.

## Data Availability

The datasets used and analyzed during the current study are available from the corresponding author upon reasonable request.

## List of abbreviations

PRISMA: Preferred Reporting Items for Systematic Reviews and Meta-analyses
MCMS: Modified Coleman Methodological Scale
RR: Relative risk
CI: Confidence Interval
AH: Anterior horn
PH: Posterior horn
M: Midbody
ACL: Anterior cruciate ligament
ACL-R: Anterior cruciate ligament reconstruction
UHMWPE: Ultra-high molecular weight polyethylene
PLLA: Poly-l-lactic-acid
